# Nomogram for Predicting Live Birth after the First Fresh Embryo Transfer in Patients with PCOS Undergoing IVF/ICSI Treatment with the GnRH-Ant Protocol

**DOI:** 10.3390/diagnostics13111927

**Published:** 2023-05-31

**Authors:** Manfei Si, Huahua Jiang, Yue Zhao, Xinyu Qi, Rong Li, Xiaoyu Long, Jie Qiao

**Affiliations:** 1 Center for Reproductive Medicine, Department of Obstetrics and Gynecology, Peking University Third Hospital, Beijing 100191, China; 2 National Clinical Research Center for Obstetrics and Gynecology, Peking University Third Hospital, Beijing 100191, China; 3 Key Laboratory of Assisted Reproduction, Peking University, Ministry of Education, Beijing 100191, China; 4 Beijing Key Laboratory of Reproductive Endocrinology and Assisted Reproductive Technology, Beijing 100191, China; 5 Beijing Advanced Innovation Center for Genomics, Beijing 100191, China; 6 Peking-Tsinghua Center for Life Sciences, Peking University, Beijing 100191, China

**Keywords:** polycystic ovary syndrome, in vitro fertilization, live birth, nomogram, prediction model

## Abstract

Polycystic ovary syndrome (PCOS) is the leading cause of anovulatory infertility. A better understanding of factors associated with pregnancy outcomes and successful prediction of live birth after IVF/ICSI are important to guide clinical practice. This was a retrospective cohort study investigating live birth after the first fresh embryo transfer using the GnRH-ant protocol in patients with PCOS between 2017 and 2021 at the Reproductive Center of Peking University Third Hospital. A total of 1018 patients with PCOS were qualified for inclusion in this study. BMI, AMH level, initial FSH dosage, serum LH and progesterone levels on the hCG trigger day, and endometrial thickness were all independent predictors of live birth. However, age and infertility duration were not significant predictors. We developed a prediction model based on these variables. The predictive ability of the model was demonstrated well, with areas under the curve of 0.711 (95% CI, 0.672–0.751) and 0.713 (95% CI, 0.650–0.776) in the training cohort and validation cohort, respectively. Additionally, the calibration plot showed good agreement between the prediction and the observation (*p* = 0.270). The novel nomogram could be helpful for clinicians and patients in clinical decision-making and outcome evaluation.

## 1. Introduction

Polycystic ovary syndrome (PCOS) is a common reproductive endocrine disorder in women of reproductive age with the prevalence of 6–20% that accounts for the majority of cases of anovulatory infertility [1]. According to its various clinical phenotypes, PCOS is categorized into four types: type I-polycystic ovary morphology and hyperandrogenemia; type II-polycystic ovary morphology and menstrual disorder; type III-hyperandrogenemia and menstrual disorder; and type IV-polycystic ovary morphology, hyperandrogenemia and menstrual disorder [2,3]. PCOS is the leading cause of anovulatory infertility [4].

Ovulation induction by the oral anti-estrogen clomiphene citrate or the aromatase inhibitor letrozole is generally considered to be the first-line drug therapy for anovulatory infertility in patients with PCOS [5]. However, when ovulation induction, including second-line gonadotropin drug treatment, fails to result in a successful pregnancy, or patients have tubal and/or male factor infertility, in vitro fertilization (IVF) treatment is an effective treatment that is recommended as the final treatment option. Because patients with PCOS are at particular risk of ovarian hyperstimulation syndrome (OHSS), the gonadotropin-releasing hormone antagonist (GnRH-ant) protocol is preferred as a safer alternative to traditional GnRH-agonist protocols because of the reduced risk of OHSS [6,7]. The GnRH-ant protocol has several strengths, including a significant reduction in the incidence of OHSS, lower dosage and shorter duration of administration, and no ‘flare-up’ effect. Therefore, the GnRH-ant protocol is increasingly favored in clinical practice for infertile patients with PCOS [6,8].

Numerous studies have identified and confirmed factors related to the clinical outcome of IVF/intracytoplasmic sperm injection (ICSI) in terms of pregnancy and/or live birth, and these factors affect nearly every stage of IVF/ICSI treatment [9,10,11,12,13,14,15,16,17,18,19,20,21,22,23,24,25]. Previous studies had predicted pregnancy outcomes after treatment in infertile couples after IVF/ICSI or ovarian stimulation–intrauterine insemination [14,15,26,27,28]. Unfortunately, few studies have evaluated how these factors can be used to predict live birth in patients with PCOS in clinical practice. If a model can be developed to predict live birth outcomes, it may facilitate better treatment stratification and outcome evaluation and provide decision-making guidance for both couples and clinicians.

The present study aimed to identify the independent factors that affect pregnancy outcomes and then develop and validate a novel nomogram model to estimate the chance of live birth in patients with PCOS undergoing their first IVF/ICSI treatment with the GnRH-ant protocol and fresh embryo transfer (ET).

## 2. Materials and Methods

### 2.1. Patients Selection

Patients with PCOS who underwent their first, fresh non-donor cycle of IVF/ICSI-ET from January 2017 to December 2021 at the Reproductive Center of Peking University Third Hospital were evaluated. The inclusion criteria were as follows: (1) patients of childbearing age between 20 and 40 years old, (2) patients with PCOS diagnosed according to the revised 2003 Rotterdam diagnostic criteria, (3) fertilization with IVF or ICSI, (4) data for only the first IVF/ICSI-ET cycle included, and (5) GnRH-ant as the controlled ovarian stimulation protocol. The exclusion criteria were as follows: (1) infertility caused by other factors such as endometriosis, adenomyosis, uterine malformations (unicornuate/bicornuate uterus, uterine septum, intrauterine adhesions), tuberculosis; (2) history of ovarian drilling, ovarian cyst or tumor removal surgery; (3) donor cycles; (4) in vitro maturation cycles; (5) chromosomal abnormality in one of the couples, and (6) preimplantation genetic testing was performed. Baseline demographics, cycle data and pregnancy outcome data were obtained from an electronic records system and medical records. The study was approved by the Ethics Committee of Reproductive Medicine at Peking University Third Hospital (No. 2021SZ-011).

### 2.2. GnRH-Ant Protocol for Ovarian Stimulation

Gonadotropin (Gn) was injected every day from the 2nd or 3rd day of the menstrual cycle. GnRH-ant was added on the 5th to 7th days after Gn administration. Transvaginal ultrasound monitoring and sex hormone level determination were performed after 3–4 days of continuous injection. The dosage was adjusted according to the ovarian response. When the diameters of 2 follicles were more than 18 mm or 3 follicles were more than 17 mm, human chorionic gonadotropin or GnRH-agonist was administered for triggering. Oocyte aspiration was performed approximately 34–36 h after triggering with a standard transvaginal ultrasound-guided approach. On the 3rd to 5th day after oocyte aspiration, high-quality embryos or blastocysts were selected for fresh ET. Oral combined vaginal progesterone was routinely used for luteal support, starting on the day of oocyte aspiration in fresh transfer cycles.

### 2.3. Primary Outcome

The primary outcome was live birth, which was defined as at least one live-born baby that survived for more than 1 month [14,27]. Baseline demographic information and laboratory markers were selected as predictor variables, including: (1) maternal and paternal age, infertility duration, infertility type, parity number, BMI (both female and male) (kg/m^2^), and antral follicle count (AFC); (2) laboratory markers included levels of basal FSH (mIU/mL), basal LH (mIU/mL), basal LH/FSH, AMH (ng/mL), triglycerides (TG) (mmol/L), total cholesterol (TC) (mmol/L), high-density lipoprotein-cholesterol (HDL-C) (mmol/L), low-density lipoprotein-cholesterol (LDL-C) (mmol/L), fasting glucose (FG) (mmol/L), fasting insulin (FINS) (μIU/mL), homeostasis model assessment-insulin resistance (HOMA-IR), serum estradiol (E_2_) on the hCG trigger day (pmol/L), serum LH on the hCG trigger day (mIU/mL), and serum P on the hCG trigger day (nmol/L); and (3) cycle data included initial FSH dose, duration of stimulation, total Gn dosage, type of fertilization, endometrial thickness, number of embryos transferred, cleavage-stage embryo or blastocyst transfer, and occurrence of live birth. HOMA-IR was calculated according to the formula “FINS × FG/22.5”. All variables were evaluated as continuous predictors for developing predictive models.

### 2.4. Prediction Model

In this model, we used all 1018 cycles. Seventy percent of the cycles were randomly selected as the training cohort to develop the prediction models. Continuous variables were expressed as the mean  ±  standard deviation (SD). Student’s *t* test and the Mann–Whitney U test were used to compare the measurement data according to data distribution between groups, and the chi-square test was performed to compare the categorical data. The results are expressed as percentage (%). Univariate logistic regression analyses were used to screen for variables that were significantly correlated with live birth in the training group. Second, predictors with a *p* value less than 0.05 and predictors that had important clinical significance were then entered into multivariate logistic regression models. Backward stepwise selection was performed with a threshold of a *p* value less than 0.1 to enter and a *p* value of less than 0.05 to remain in the final model. A nomogram was delineated to visualize the established model based on the results of multivariate logistic regression analysis and by using the package of rms in R, version 4.0.3. The predictive efficacy of the model was assessed by means of discrimination and calibration. Discrimination describes the ability of the model to distinguish between those patients who have the outcome of live birth and those who do not, which was assessed by means of the area under the receiver operating characteristic (ROC) curve (AUC). ROC curves show the model sensitivity and specificity. Calibration describes the consistency between the model-predicted probabilities and the observed probabilities of a live birth, which was assessed by means of the Hosmer–Lemeshow test, with a *p* value of more than 0.05 indicating a good fit of the data. The remaining 30% of the cycles were used as an internal validation cohort. IBM SPSS Statistics (version 25) and R software (version 1.3) were used for data analysis, further nomogram establishment and AUC measurements. A standard *p* value less than 0.05 was considered statistically significant in all tests.

## 3. Results

### 3.1. Characteristics of the Training and Validation Cohorts

**Figure 1:**
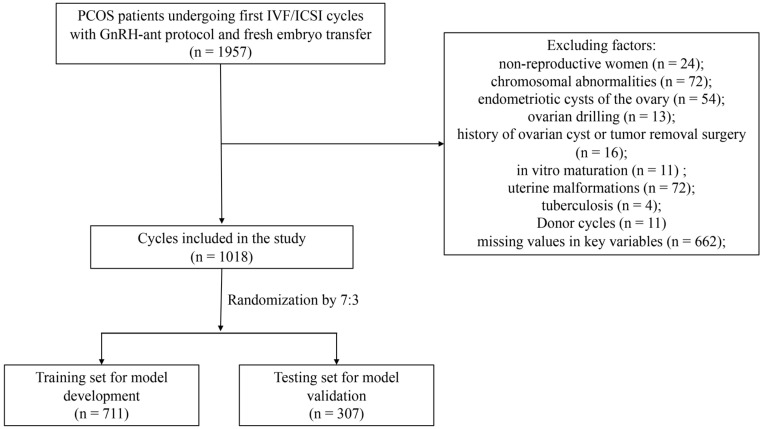
Flow chart of patient selection. PCOS, polycystic ovary syndrome; IVF/ICSI, in vitro fertilization/intracytoplasmic sperm injection.

In total, 1018 patients with PCOS who had undergone IVF/ICSI cycles with fresh ET using the GnRH-ant protocol from January 2017 to December 2021 were included in this study (Figure 1). The baseline characteristics of the participants are shown in Table 1. The average maternal age was 31.41 ± 4.04 years, and the number of oocytes retrieved in the first cycle was 16.65 ± 7.80. A total of 214 patients (30.1%) had live births in the training cohort. The overall live birth rate for the validation cohort was 26.7%. The demographic variables of both the training and validation cohorts were comparable with no significant difference, as were the overall pregnancy outcomes, including clinical pregnancy rates, miscarriage rates and live birth rates.

### 3.2. Logistic Regression Analysis

Univariate logistic regression analysis was performed to screen the factors significantly associated with clinical pregnancy and live birth in the training group (Table 2). As live birth was considered the final and most clinically significant outcome of IVF/ICSI, we performed multivariate logistic regression using predictors with a *p* value less than 0.05 and those had important clinical significance. As shown in Table 3, after backward stepwise selection to remove potential redundancy, BMI (OR = 0.929, 95% CI: 0.887–0.974), AMH level (OR = 0.950, 95% CI: 0.908–0.994), initial FSH dosage (OR = 0.992, 95% CI: 0.985–0.999), serum LH level on the hCG trigger day (OR = 1.101, 95% CI: 1.032–1.175), serum P level on the hCG trigger day (OR = 1.243, 95% CI: 1.011–1.529), and endometrial thickness (OR = 1.383, 95% CI: 1.241–1.542) remained significantly associated with live birth in multivariate analysis.

### 3.3. Building the Nomogram Predictive Model in the Training Cohort

**Figure 2:**
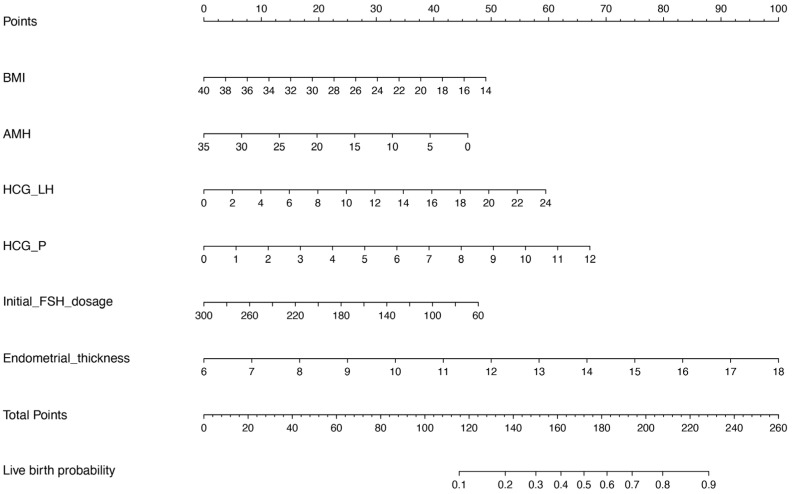
Nomograms for predicting live birth. BMI, body mass index; AMH, anti-Müllerian hormone; HCG_LH, serum LH level on the hCG trigger day; HCG_P, serum P level on the hCG trigger day.

Based on the results of the multivariate logistic regression analysis, we constructed a nomogram model with the significant risk factors for BMI, AMH level, initial FSH dosage, serum LH and P levels on the hCG trigger day, and endometrial thickness to predict the probability of live birth (Figure 2). The final score was calculated by adding up the score of each item and then obtaining the predicted possibility of live birth by drawing straight down to the live birth axis. For example, for a female with BMI = 24 kg/m^2^ (30 points), AMH level = 5 ng/mL (40 points), serum LH level on the hCG trigger day = 3 mIU/mL (7.5 points), serum P level on the hCG trigger day = 1 nmol/L (6 points), initial FSH dosage = 150 IU/day (30 points) and endometrial thickness = 9 mm (25 points), the total points is 138.5, and the suspected live birth rate is approximately 20%. This calculated value could be used in decision-making for treatment plans and patient counseling.

### 3.4. Validation of the Predictive Model in the Validation Cohort

**Figure 3:**
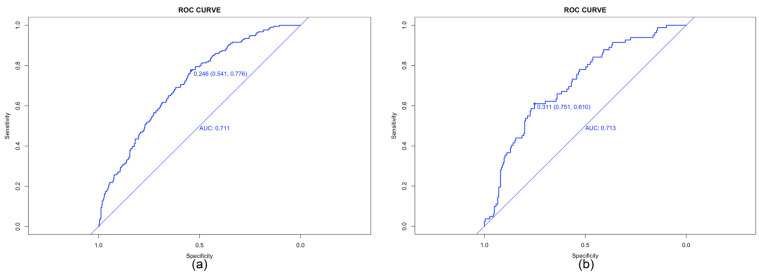
Receiver operating characteristic (ROC) curves for the prediction of live birth in the training set (**a**) and validation set (**b**). AUC, area under ROC curve.

**Figure 4:**
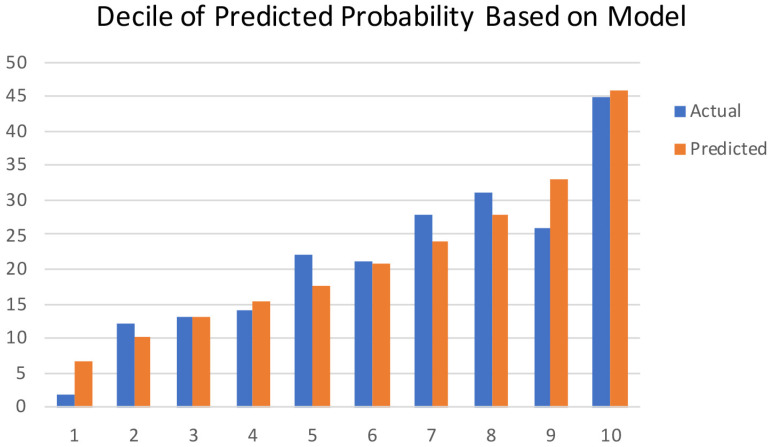
Calibration plots show the relationship between the predicted probabilities based on the nomogram and the actual values of the cohort. The x-axis represents deciles of predicted risk, and the y-axis represents the predicted and actual probability of live birth. The Hosmer–Lemeshow chi-square was 9.933 (*p* = 0.270).

The model performance was evaluated by its discrimination and calibration abilities. As shown in Figure 3, ROC curves were generated, and AUCs of 0.711 (95% CI, 0.672–0.751) and 0.713 (95% CI, 0.650–0.776) were calculated for the training and validation cohorts, respectively; these results indicated an acceptable discrimination of the model in clinical practice [29]. The Hosmer–Lemeshow test was performed to evaluate the calibration of the model. The Hosmer–Lemeshow chi-square statistic was 9.933, and the calibration plot is presented in Figure 4, which showed good agreement between the predicted chances and the observed chances (*p* = 0.270).

## 4. Discussion

Recently, a large number of couples have been diagnosed with infertility, of which 80% of anovulatory infertility cases were PCOS [30,31]. A better understanding of factors associated with pregnancy outcomes and successful prediction of live birth after IVF/ICSI are important to guide clinical practice and to counsel patients effectively. In this study, a novel nomogram prediction model was developed and validated using a large cohort of patients with PCOS at Peking University Third Hospital. This nomogram, based on BMI, AMH level, initial FSH dosage, serum LH and P levels on the hCG trigger day, and endometrial thickness, can be used to predict the probability of a successful live birth in patients with PCOS undergoing fresh IVF with the GnRH-ant protocol and can be used by clinicians and patients in treatment decision-making.

Age as an important predictor of clinical outcomes is not surprising, so we also discussed the effects of paternal and maternal age on the pregnancy outcomes of IVF in this study. Increasing maternal age is linked with poorer oocyte quality and decreased fertility, but studies on the effect of male age on infertility treatments are poorly explored. Horta et al. found that both female age and male age were negatively associated with the chance of live birth and clinical pregnancy in IVF/ICSI cycles (*p* < 0.001) [10]. McPherson et al. also indicated strong negative associations for maternal age (*p* < 0.001) and paternal age (*p* = 0.04) with live births [11]. However, the present study did not find a significant association between couple age and live birth. The possible reason is that patients with PCOS have a high ovarian reserve so that age does not add to the value of the final model construction.

Increasing evidence shows that female obesity is associated with adverse pregnancy outcomes following IVF procedures [32,33]. Sermondade et al. demonstrated a decreased probability of live birth following IVF in obese (BMI ≥ 30 kg/m^2^) women compared to normal weight (BMI 18.5–24.9 kg/m^2^) women with an OR of 0.85 (95% CI, 0.82–0.87), which included a total of 21 studies [12]. However, other studies have reported that BMI was not associated with IVF/ICSI pregnancy outcomes, including the clinical pregnancy rate and live birth rate, but was negatively related to pregnancy complications such as gestational diabetes mellitus and gestational hypertension [13]. The results of our study proved that the live birth rate in overweight and obese patients with PCOS was significantly decreased. Additionally, in multivariate logistic regression analysis adjusted for age, BMI, infertility duration, AFC, fasting serum glucose level, stimulation type, basal FSH level, basal LH level and basal E_2_ level, a one kg/m^2^ increase in female BMI was associated with a 6.8% decreased possibility of live birth (OR 0.932, 95% CI 0.893–0.971; Table 1). In addition, between 40% and 80% of patients with PCOS are overweight or obese, and at an increased risk of developing gestational diabetes mellitus [34,35,36]. Quaresima et al. previously examined pre-pregnancy/pregnancy related risk factors that associated to stillbirth and found that maternal overweight or obesity (50.9%) and gestational diabetes mellitus (15%) were the most common risk factors [37]. Hence, PCOS patients that are overweight or obese have a lower live birth rate as well as a higher risk of stillbirth. It is highly recommended that all women with PCOS should maintain a healthy weight in order to improve their overall health and pregnancy outcomes.

Patients with shorter infertility duration and secondary infertility had a higher chance of live birth [15,26,38,39]. Similarly, infertility type and duration were significant predictors of live birth in this study (*p* < 0.05). In the multivariate logistic regression analysis, they were not significantly associated with pregnancy outcomes. It is easy to understand that couples diagnosed with secondary infertility had a history of pregnancy, which means that they at least have the ability to conceive, and those with a shorter duration of infertility were more likely to be affected by fewer infertility factors than those with a longer duration.

The association between serum AMH level and treatment outcomes of IVF/ICSI has also been discussed in many studies. Brodin et al. found that the AMH level was strongly associated with live birth rates after IVF-ICSI, especially in patients with polycystic ovaries [20]. Li et al. investigated the relationship between AMH level and live birth outcome after IVF procedures in 27,029 women and suggested that AMH was associated with live birth in advanced-age women but not in women with diminished ovarian reserve or younger age [18]. Guo et al. found that AMH had a significant negative association with fresh live birth rate and clinical pregnancy rate only in patients with PCOS < 30 years old [19]. Although AMH was not significantly correlated with live birth in univariate analyses in our study, it had a negative correlation in the multivariate analysis and was included in the final prediction model of live birth.

With a high incidence of glucose and lipid metabolism disorders in patients with PCOS, this study also explored whether these metabolic indicators, including TG, TC, HDL-C, LDL-C, FG, FINS and HOMA-IR, had effects on live birth outcomes in infertile patients with IVF. In the univariate analysis, we only found that the LDL-C level had a significantly negative impact on live birth (*p* = 0.034), which was the same as in a previous study [40]. However, HOMA-IR, which indicated insulin resistance, was not associated with live birth. However, not all patients had their insulin level checked. A total of 746 of 1019 patients had HOMA results, which may have induced selection bias in our study. Further large-scale studies are needed to identify the association between metabolism disorders and pregnancy outcomes after IVF/ICSI in patients with PCOS.

In addition to known predictors, such as age, BMI, AMH level, serum hormone levels on the hCG trigger day, including levels of LH, P, and E_2_, were found to be associated with the number of retrieved oocytes and pregnancy outcomes during IVF-ET procedures [21,22,41,42,43,44]. Benmachiche et al. identified that low serum LH levels on the hCG trigger day are associated with reduced ongoing pregnancy and live birth rates and increased early miscarriage rates, and a serum LH level of 1.60 mIU/mL was considered a threshold [21]. Venetis et al. found that live birth rates were significantly decreased in the group with P > 1.5 ng/mL on the day of hCG during a multivariate analysis [43], which was the same as Santos-Ribeiro’s study [45]. In addition, it was reported that both low (≤0.5 ng/mL) and high (>1.5 ng/mL) levels of P on the hCG trigger day were significantly associated with lower live birth rates [22,46]. To this end, our findings of a positive correlation between LH and P levels on the hCG trigger day and live birth (*p* < 0.001), also in the multivariate analysis, are not surprising.

It is widely recommended that an endometrial thickness greater than 7 mm is considered to be ready for ET [24,47]. Lv et al. conducted a retrospective cohort study including 15,012 ART cycles and found that the live birth rate increased with increasing endometrial thickness and reached a plateau when endometrial thickness was 11 mm or thicker [23]. Our finding of a significant positive interaction between endometrial thickness and live birth (*p* = 0.023) indicated the prognostic value of endometrial thickness on live birth in clinical practice for patients with PCOS undergoing fresh transfer.

Whether blastocyst transfer increases the pregnancy outcomes compared with cleavage-stage ET is controversial. Wang et al. suggested that live birth after fresh IVF/ICSI is significantly improved following blastocyst transfer compared to cleavage-stage ET (OR 1.77; 95% CI, 1.32–2.37) [25]. Glujovsky et al. included 32 RCTs and found that the live birth rate following fresh transfer was higher in the blastocyst-stage transfer group (OR 1.27; 95% CI, 1.06–1.51) [48]. However, additional evidence showed no superiority of blastocyst transfer over cleavage-stage ET in terms of live birth in clinical practice [49]. The present analysis did not reveal that blastocyst transfer was associated with an increased live birth rate compared with cleavage-stage ET, partly because single blastocyst transfer was preferred, while the number of cleavage-stage ETs was mainly two.

To our knowledge, this is the first prediction model for live birth in patients with PCOS undergoing their first fresh IVF/ICSI cycles with good discrimination and the best concordance between the prediction and actual observation, thus guaranteeing its reliability. Inevitably, our study has some limitations. One is that we were unable to account for some routine confounders, such as smoking status and alcohol intake. Second, this was a retrospective study, and there were 273 patients with missing insulin data, which cannot fully prevent potential biases such as selection bias. Third, the data were obtained from our single center, and evidence from other centers is needed for external validation of this prediction model. Furthermore, this model was limited to the use of fresh cycles only, while patients with PCOS had high cycle cancellation rates for OHSS; thus, further studies should concentrate on the cumulative pregnancy rate after fresh and frozen-thawed ET cycles.

## 5. Conclusions

In conclusion, in addition to identifying risk factors associated with pregnancy outcomes, we also developed a nomogram model to predict live birth after IVF/ICSI for patients with PCOS in this large prospective study. BMI, AMH level, initial FSH dosage, serum LH level on the hCG trigger day, serum P level on the hCG trigger day and endometrial thickness were independent significant predictors of live birth. The proposed model shows good predictive discrimination and calibration. It can be conveniently used to improve the individualized prediction of live birth in patients with PCOS before embryo transfer in the IVF/ICSI procedure, to help clinicians make informed clinical decisions and to provide personalized treatment plans that can improve patients’ pregnancy outcomes. We hope that this prediction model will help patients with PCOS in their counseling process and decision-making before they embark on their first treatment cycle. It also has the potential to drive the development of new treatments and therapies for PCOS patients by identifying factors that contribute to successful pregnancy outcomes. This could lead to significant improvements in the quality of care for PCOS patients and contribute to the overall advancement of the field of fertility treatments.

## Figures and Tables

**Table 1:** Patient and IVF cycle characteristics of training set and testing set.

Characteristics	Training Set (%)	Testing Set (%)	*p*
No. of cases	711	307	
Maternal age (years)	30.33 ± 3.41	30.43 ± 3.48	0.680
Paternal age (years)	31.86 ± 4.39	31.86 ± 4.26	0.989
Infertility duration (years)	3.49 ± 2.40	3.82 ± 2.42	0.047
Infertility type			
Primary infertility	485 (68.2%)	213 (69.4%)	0.713
Secondary infertility	226 (31.8%)	94 (30.6%)	
Parity number			
Nulliparous	685 (96.3%)	290 (94.5%)	0.171
Parous	26 (3.66%)	17 (5.5%)	
Maternal BMI (kg/m^2^)	25.19 ± 3.96	25.56 ± 4.07	0.179
<25	353 (49.6%)	154 (50.2%)	0.509
25–30	268 (37.7%)	107 (34.9%)	
≥30	90 (12.7%)	46 (15.0%)	
Paternal BMI (kg/m^2^)	25.79 ± 4.29	26.07 ± 4.39	0.334
bFSH (mIU/mL)	6.03 ± 1.62	6.18 ± 1.76	0.190
bLH (mIU/mL)	5.06 ± 3.12	5.25 ± 3.16	0.370
LH/FSH	0.85 ± 0.50	0.87 ± 0.56	0.590
AMH (ng/mL)	7.31 ± 4.08	6.89 ± 3.82	0.125
AFC (n)	24.42 ± 5.41	24.42 ± 5.83	0.992
TG (mmol/L)	1.60 ± 1.07	1.62 ± 1.14	0.747
TC (mmol/L)	4.61 ± 0.83	4.64 ± 0.77	0.716
HDL-C (mmol/L)	1.23 ± 0.27	1.27 ± 0.63	0.277
LDL-C (mmol/L)	2.95 ± 0.73	2.95 ± 0.72	0.934
FG (mmol/L)	4.99 ± 0.70	5.05 ± 0.89	0.253
FINS (μIU/mL)	13.35 ± 10.63	14.30 ± 8.14	0.236
HOMA-IR	3.01 ± 2.96	3.27 ± 2.12	0.245
Initial FSH dosage (IU/day)	145.68 ± 28.15	148.94 ± 31.10	0.100
Duration of stimulation (days)	10.77 ± 2.24	10.68 ± 2.14	0.559
Total Gn dosage (IU)	1908.22 ± 817.42	1916.98 ± 718.79	0.871
Serum E_2_ level on the hCG trigger day (pmol/L)	1588.42 ± 3470.95	1605.64 ± 3506.29	0.942
Serum LH level on the hCG trigger day (mIU/mL)	2.76 ± 2.79	2.71 ± 2.58	0.751
Serum P level on the hCG trigger day (nmol/L)	1.74 ± 0.89	1.79 ± 0.92	0.480
Number of retrieved oocytes (n)	10.62 ± 4.99	10.66 ± 4.36	0.891
Endometrial thickness	10.32 ± 1.69	10.10 ± 1.62	0.060
Type of fertilization			
Routine	536 (75.4%)	219 (71.3%)	0.132
ICSI	167 (23.5%)	80 (26.1%)	
Half-ICSI	8 (1.1%)	8 (2.6%)	
2PN	6.30 ± 3.36	6.62 ± 3.49	0.179
Number of transplantable embryos	3.92 ± 2.50	4.15 ± 2.58	0.179
Days of embryo transfer			
D3-ET	683 (96.1%)	294 (95.8%)	0.825
D5/6-ET	28 (3.9%)	13 (4.2%)	
Number of embryo transfer			
1	76 (10.7%)	34 (11.1%)	0.856
2	635 (89.3%)	273 (88.9%)	
Clinical pregnancy			
Yes	309 (43.5%)	115 (37.5%)	0.075
No	402 (56.5%)	192 (62.5%)	
Miscarriage			
Yes	95 (30.7%)	33 (28.7%)	0.683
No	214 (69.3%)	82 (71.3%)	
Live birth			
Yes	214 (30.1%)	82 (26.7%)	0.275
No	497 (69.9%)	225 (73.3%)	

Abbreviations: BMI, body mass index; bFSH, basal follicle-stimulating hormone; bLH, basal luteinizing hormone; AMH, anti-Müllerian hormone; AFC, antral follicle count; TG, triglyceride; TC, total cholesterol; HDL-C, high-density lipoprotein-cholesterol; LDL-C, low-density lipoprotein-cholesterol; FG, fasting glucose; FINS, fasting insulin; HOMA-IR, homeostasis model assessment-insulin resistance; Gn, gonadotropin; E_2_, estradiol; P, progesterone; ICSI, intracytoplasmic sperm injection; PN, pronucleus; ET, embryo transfer. Continuous variables are expressed as the mean ± SD; categorical variables are expressed as percentages.

**Table 2:** Univariate analysis results summary for pregnancy outcomes of clinical pregnancy and live birth.

Variables	Clinical Pregnancy		Live Birth	
	OR (95% CI)	*p*	OR (95% CI)	*p*
Maternal age (years)	0.996 (0.954, 1.040)	0.859	0.967 (0.923, 1.014)	0.170
Paternal age (years)	1.008 (0.974, 1.042)	0.654	0.998 (0.962, 1.035)	0.908
Infertility duration (years)	0.915 (0.858, 0.976)	0.007	0.932 (0.868, 1.000)	0.049
Infertility type				
Primary infertility	1 (reference)	-	1 (reference)	-
Secondary infertility	0.795 (0.577, 1.095)	0.161	0.638 (0.445, 0.914)	0.014
Parity number				
Nulliparous	1 (reference)		1 (reference)	-
Parous	0.952 (0.431, 2.104)	0.904	0.851 (0.352, 2.055)	0.719
Maternal BMI (kg/m^2^)	0.957 (0.921, 0.994)	0.022	0.932 (0.893, 0.971)	0.001
<25	1 (reference)	-	1 (reference)	-
25–30	0.654 (0.473, 0.903)	0.010	0.645 (0.455, 0.914)	0.014
≥30	0.710 (0.443, 1.136)	0.153	0.488 (0.281, 0.847)	0.011
Paternal BMI (kg/m^2^)	0.970 (0.936, 1.004)	0.086	0.966 (0.929, 1.004)	0.075
bFSH (mIU/mL)	0.950 (0.866, 1.041)	0.271	0.938 (0.848, 1.037)	0.208
bLH (mIU/mL)	1.014 (0.968, 1.064)	0.552	1.005 (0.955, 1.058)	0.837
LH/FSH	1.203 (0.897, 1.614)	0.217	1.130 (0.827, 1.544)	0.443
AMH (ng/mL)	0.962 (0.927, 0.999)	0.045	0.968 (0.929, 1.008)	0.118
AFC (n)	1.005 (0.978, 1.033)	0.723	1.007 (0.978, 1.037)	0.629
TG (mmol/L)	0.875 (0.753, 1.016)	0.875	0.848 (0.714, 1.007)	0.060
TC (mmol/L)	0.824 (0.685, 0.991)	0.040	0.846 (0.692, 1.034)	0.102
HDL-C (mmol/L)	1.247 (0.709, 2.192)	0.443	1.518 (0.83, 2.775)	0.175
LDL-C (mmol/L)	0.761 (0.616, 0.940)	0.011	0.780 (0.619, 0.982)	0.034
FG (mmol/L)	0.964 (0.777, 1.197)	0.742	0.852 (0.658, 1.103)	0.224
FINS (μIU/mL)	1.009 (0.992, 1.027)	0.285	1.002 (0.984, 1.019)	0.856
HOMA	1.037 (0.971, 1.108)	0.273	1.012 (0.952, 1.075)	0.708
Initial FSH dosage (IU/day)	0.996 (0.990, 1.001)	0.131	0.994 (0.988, 1.000)	0.056
Duration of stimulation (days)	0.931 (0.870, 0.997)	0.042	0.926 (0.859, 1.000)	0.049
Total Gn dosage (IU)	1.000 (1.000,1.000)	0.057	1.000 (1.000,1.000)	0.023
Serum E_2_ level on the hCG trigger day (pmol/L)	1.000 (1.000, 1.000)	0.228	1.000 (1.000, 1.000)	0.116
Serum LH level on the hCG trigger day (mIU/mL)	1.149 (1.082, 1.219)	<0.001	1.139 (1.076, 1.206)	<0.001
Serum P level on the hCG trigger day (nmol/L)	1.252 (1.050, 1.491)	0.012	1.416 (1.176, 1.705)	<0.001
Endometrial thickness	1.362 (1.235, 1.501)	<0.001	1.412 (1.271, 1.568)	<0.001
Type of fertilization				
Routine	1 (reference)	-	1 (reference)	-
ICSI	0.873 (0.614, 1.242)	0.451	0.967 (0.661, 1.416)	0.864
Half-ICSI	2.119 (0.501, 8.955)	0.307	2.329 (0.575, 9.428)	0.236
Days of embryo transfer				
D3-ET	1 (reference)	-	1 (reference)	-
D5/6-ET	0.605 (0.270, 1.356)	0.222	0.926 (0.401, 2.137)	0.857
Number of embryos transferred				
1	1 (reference)	-	1 (reference)	-
2	1.450 (0.884, 2.379)	0.142	1.699 (0.955, 3.024)	0.071

Abbreviations: BMI, body mass index; bFSH, basal follicle-stimulating hormone; bLH, basal luteinizing hormone; AMH, anti-Müllerian hormone; AFC, antral follicle count; TG, triglyceride; TC, total cholesterol; HDL-C, high-density lipoprotein-cholesterol; LDL-C, low-density lipoprotein-cholesterol; FG, fasting glucose; FINS, fasting insulin; HOMA-IR, homeostasis model assessment-insulin resistance; Gn, gonadotropin; E_2_, estradiol; P, progesterone; ICSI, intracytoplasmic sperm injection; ET, embryo transfer. Continuous variables are expressed as the mean ± SD; categorical variables are expressed as percentages.

**Table 3:** Multivariate logistic regression for live birth.

Variables	OR (95% CI)	*p*
BMI (kg/m^2^)	0.929 (0.887, 0.974)	0.002
AMH (ng/mL)	0.950 (0.908, 0.994)	0.027
Initial FSH dosage (IU/day)	0.992 (0.985, 0.999)	0.028
Serum LH level on the hCG trigger day (mIU/mL)	1.101 (1.032, 1.175)	0.004
Serum P level on the hCG trigger day (nmol/L)	1.243 (1.011, 1.529)	0.039
Endometrial thickness	1.383 (1.241, 1.542)	<0.001

Abbreviations: BMI, body mass index; AMH, anti-Müllerian hormone; FSH, follicle-stimulating hormone; LH, luteinizing hormone; P, progesterone.

## Data Availability

The data presented in this study are available on request from the corresponding author.
